# Genetic Variation between Triploid and Diploid *Clarias gariepinus* (Burchell, 1822) Using RAPD Markers

**DOI:** 10.3390/vetsci8050075

**Published:** 2021-05-04

**Authors:** Jalil Normala, Victor Tosin Okomoda, Azizul Alim Mohd, Asma Ariffin Nur, Ambok Bolong Abol-Munafi, Shahreza Md Sheriff

**Affiliations:** 1 Faculty of Food Science and Fisheries, Universiti Malaysia Terengganu, Kuala Nerus 21030, Malaysia; normalaj@gmail.com (J.N.); azizual@umt.edu.my (A.A.M.); asma@umt.edu.my (A.A.N.); 2 Department of Fisheries and Aquaculture, College of Forestry and Fisheries, University of Agriculture, Makurdi P.M.B. 2373, Nigeria; 3 Higher Institution Centre of Excellence (HICoE), Institute of Tropical Aquaculture and Fisheries Research (AQUATROP), Universiti Malaysia Terengganu, Kuala Nerus 21030, Malaysia

**Keywords:** African catfish, SCAR, polyploidy, genetic distance

## Abstract

This study was designed to examine the use of RAPD markers in discriminating triploid and diploid African catfish *Clarias gariepinus* (Burchell, 1822). Following a routine technique, triploidy was induced by cold shock and confirm by erythrocyte measurement in *C. gariepinus*. Thereafter, 80 RAPD markers were screened; out of which, three showed the highest percentage of polymorphism (i.e., OPB 16 = 71.43%; OPC 14 = 61.9%; OPD 12 = 75%). The results obtained showed genotype differences between triploid and diploid without overlapping. However, the development of a Sequence Characterized Amplified Region (SCAR) marker was not achievable because progenies of triploid and diploid *C. gariepinus* could not be differentiated based on a specific fragment. Consequently, the genetic distance showed high similarities for both treatments and the UPGMA-generated dendrogram could not separate the treatments into two distinct clusters. It was concluded that RAPD makers cannot be used to separate the ploidy status of fishes.

## 1. Introduction

Triploidy induction during breeding is one of the genetic manipulation methods used to alter the chromosome number of many cultured organisms in an attempt to improve performance characteristics [1]. Triploid animals are produced by inhibiting the release of the second polar body through the application of physical or chemical shock shortly after fertilization [2]. The consequences of triploidy induction in aquaculture are numerous and range from better growth performance to the production of sterile fish [3]. As a result, triploid fish can attain market size earlier than diploids and prevent prolific breeding in fishes with precocious sexual maturity/uncontrolled reproduction [4]. Sterility in triploids is an important management tool in preventing contamination of a local gene pool [5]. Identification of triploids, however, is paramount to the management of the progenies in captivity or escapees into the natural environment [6,7]. Several methods have been used to characterize and differentiate diploid from triploid fishes, however, these methods are not without their pros and cons.

Some studies have used erythrocyte size as a simpler index of differentiating triploid organisms from their diploid counterparts [5,7,8]. However, significant size distribution overlap between triploid and diploid erythrocytes has raised notable concern about the accuracy of the erythrocyte characterization method [9]. Many studies have also reported the use of karyotyping to determine triploid fish [9]. Aside from the tedious need to optimize many factors for this method [10,11,12], it also requires that the fish be sacrificed, hence, it is not suitable for large scale characterization of triploid fish [6]. The electrophoresis of proteins [13], DNA content determination with flow cytometry [14], and the numbers of nucleolar organizing regions (NORs) per chromosome pair [15] are other widely used methods; however, they require specific equipment and expensive materials [16]. The search for a more suitable, rapid, inexpensive, and accurate method for large scale triploid identification (without sacrificing the fish) is still needed.

DNA markers have been used and applied widely in the aquaculture sector [17]. Markers such as RAPD have been successfully applied in species identification, gender determination [18], and hybrid identification [19]. Genetic markers have found a pride of place in diversity and resource analysis of aquaculture stock. However, despite the use of DNA markers in several aspects of the aquaculture sector, to the knowledge of the authors, genetic analysis of triploid and diploid fishes has not been reported to date. The application of RAPD can lead to the development of the Sequence Characterized Amplified Region (SCAR) marker. SCAR is one of the stable markers, generally derived from RFLP, RAPD, and AFLP markers [20]. It is relatively easy and can be used to distinguish strain from the base population [21,22]. This is not to say RAPD patterns reproducibility are not without some challenges as it is dependent on factors such as PCR conditions, DNA quality/concentration, PCR components concentrations, etc. However, it is hypothesized that if SCAR was successfully developed for triploid fishes, it can allow for quick and robust discrimination between triploid and diploid fish.

The possibility of identifying SCAR markers in triploid fishes is based on the assumption that the application of temperature shock protocol (i.e., altered environmental factor) at a very sensitive stage of embryonic development may have resulted in mutagenic effects or genetic changes in the fish group while doubling the chromosome number [5]. Hence, RAPD markers could help identify these anomalies that would be evident only in the triploid fishes as is the case of a distinct strain of a population. The African catfish *Clarias gariepinus* is not just an important aquaculture species because of its popularity of culture around the world [23] but considered one of the best animal models for biological studies [24]. The production of triploid African catfish has been previously reported [8]. In this study, we attempt to determine the genetic variation between triploid and diploid progenies of *C. gariepinus* using RAPD makers.

## 2. Materials & Methods

### 2.1. Triploidy Induction of the Catfish

Triploid fish were obtained following the breeding and induction method described by Normala et al., [6,7]. In brief, three pairs of sexually matured broodstocks of *C. gariepinus* (about 1000 g) were injected with Ovaprim^®^ (at 0.5 mL kg^−1^) and maintained in separate tanks for a latency period of 10 h. Eggs were collected from all the females by striping while the males were euthanized before lacerating their abdominal cavity to obtain the testis. Fertilization was then achieved by mixing the pooled eggs with the milt from all the males and activation of the sperm with saline water (5% saline). The fertilized eggs were quickly divided into six places for the two treatments with three replicates intended for the study. Thereafter, cold shock (i.e., 5 °C water bath for 20 min) was applied to the eggs for triploidization at approximately 3 min after fertilization. Both the cold treated batch of eggs and the control eggs were incubated in triplicates 100 L tanks with continuous aeration. Upon hatching, the fry was maintained for three months (feeding initially with *Artemia nauplii ad libitum* and later with catfish starter diet). At this age, the ploidy status of the juveniles was confirmed following the triploid erythrocyte exclusive range (i.e., erythrocyte main axis of 11.9–14.9 μm) used by Normala et al., [6,7], Hassan et al., [5] and Okomoda et al., [2].

### 2.2. DNA Extraction

DNA analysis was done only after the triploidy status of the juveniles to be used was confirmed (i.e., 30 juvenile each of the triploid and diploid progenies). DNA was extracted using a DNA extraction kit (DNA Purification Kit Fermentas, Waltham, MA, USA) following the manufacturer’s label. In brief, approximately, 50 to 100 mg of fin samples were transferred into a 1.5 mL tube and mixed with 400 μL of lysis solution. The fin samples were cut into small pieces and incubated at 65 °C for 15 min. After that, 600 μL of chloroform was added. The sample was then mixed by inversion 3 to 5 times and centrifuged at 10,000 rpm for 2 min. The upper aqueous phase containing DNA was transferred into a new tube and 800 μL of freshly prepared precipitation solution of 720 μL of sterile deionized water with 80 μL of supplied 10× concentrated solution were added. The mixture was mixed by inversion at room temperature for 1 to 2 min and centrifuged at 100,000 rpm for 2 min.

The supernatant was removed, and the DNA pellet was dissolved in 100 μL of 1.2 M NaCl solution. After that 1 μL of RNase was added and mixed by tapping and a short spin of centrifugation. The sample was incubated for 30 min in the heating block. Then, 300 μL of 95% cold ethanol was added and the DNA was precipitated overnight at −20 °C before centrifugation at 13,200 rpm for 4 min. The ethanol was poured off and the pellet was washed with 300 μL of 70% cold ethanol. Then the DNA was centrifuged at 13,200 rpm for 4 min. The ethanol was removed, and the DNA pellet was dried for 5 min at room temperature. The DNA pellet was dissolved in 50 μL of sterilized deionized water.

Lastly, the concentration and purity (A_260/280_) of DNA were measured by using a UV spectrometer (Eppendorf, BioPhotometer, Hamburg, Germany) and confirmed to be within the ratio of good quality of the DNA (i.e., 1.8–2.0). Thereafter, a 50 ng/μL concentration DNA stock was prepared for each sample and used for PCR amplification.

### 2.3. PCR Amplification

For this experiment, the PCR condition was optimized. Hence, upon optimization, PCR was done for PCR mixture containing 1.0 μL of a single primer, 2.5 μL 10× PCR buffer, 2.5 μL MgCl, 0.5 μL of each dNTPs, 17.3 μL sterilized deionized water, 0.2 μL of *Taq* DNA polymerase (Promega, Madison, WI, USA) and 1.0 μL DNA (100 ng). Then, PCR amplification was performed in the DNA Engine Peltier Thermal Cycler (Bio-Rad, Hercules, CA, USA) as follows: initial denaturing for 3 min at 94 °C, followed by 40 cycles at 94 °C for 1 min, 36 °C for 2 min, 72 °C for 2 min and a final extension at 72 °C for 4 min. The screening was done using 3 samples for each treatment for each one of the 80 primers (i.e., OPA 01-20; OPB 01-20; OPC 01-20; OPD 01-20). The primers which showed the highest polymorphic pattern after gel electrophoresis were selected for analysis of all the samples from both treatments (i.e., 30 samples each for the triploid and diploid progenies). The gel electrophoresis was done using a 1% agarose gel to determine the quality of DNA and 2% gel to determine the PCR product. The gel was run at 95 V for 80 min. The agarose gel was stained using Ethidium bromide (Eth Bro, Merck KGaA, Darmstadt, Germany) for 2 min. Then, the gel was rinsed with distilled water for 20 min. The gel was viewed in an image documentation system (BIO-RAD, Hercules, CA, USA) for analysis under a UV light and printing the result on a thermapaper.

### 2.4. Genetic Data Analysis

The 80 primers were screened using three samples from both treatments (triploid and diploid). From the gel result, the percentage of polymorphism was gotten using the formulae below:

(1) $$\mathrm{Percentage}\;\mathrm{of}\;\mathrm{polymorphism}=\left(\frac{\mathrm{Number}\;\mathrm{of}\;\mathrm{polymorphic}\;\mathrm{fragments}}{\mathrm{Number}\;\mathrm{of}\;\mathrm{total}\;\mathrm{amplified}\;\mathrm{fragments}}\right)\times \;100$$

The three primers that showed the highest polymorphic percentage were chosen for genetic analysis. The three primers were used to amplify 30 samples each for both treatments (triploid and diploid). The position of the RAPD bands in each electrophoresis lane was marked as bp (base-pair) unit by comparing the sample band with the standard band 100 bp (DNA marker) at both margin lanes. The profile was then analyzed to see the band for similarity and variation between triploid and diploid progenies. Data were recorded as 1 (present) and 0 (absent). The analysis was conducted by using the NTSYS-pc version 2.10 computer programs. The similarity index was then used to construct a similarity tree (dendrogram) showing the relationships among accessions using the Unweight pair group method of arithmetic average (UPGMA) analysis.

## 3. Results and Discussion

The RAPD profiles of individuals from the triploid and diploid fish samples were obtained from the amplification of all the 80 primers (Table 1). The results showed that the fragment size varied from 180 bp to 3000 bp. Among the 80 primers that were used in this study, only three primers showed a high percentage of polymorphism; namely, OPB 16 (71.43%), OPC 14 (61.90%), and OPD 12 (75.00%). These three primers were chosen for genetic analysis of all the samples of the triploid and diploid fish. This is in consonant with several studies that earlier used RAPD markers for biological research [25,26,27]. Analysis of the profile showed that the number of fragments obtained from OPB 16, OPC 14, and OPD 12 was 20, 35, and 24, respectively, corresponding to a polymorphic percentage of 80%, 91.4%, and 91.67%. Each primer produced a different number of fragments and profiles (Table 2). Most of the selected RAPD primers in previously reported studies had also shown polymorphism between 61.53 to 91.4% [20,28].

The results of this study showed genotype differences between triploid and diploid African catfish using the RAPD method. Of the 48 genotypes observed in the OPB 16, a total of 22 genotypes were belonging to the diploid progenies while the remaining 26 genotypes belong to the triploid (Table 3a). In OPC 14 however, a total of 59 genotypes were observed with 30 belonged to the diploid and 28 belonging to the triploid progenie (Table 3b). OPD 12, on the other hand, had 52 genotypes in total; 30 of the genotypes were found in diploid while 22 genotypes were in the triploid (Table 3c). Until now genetic analysis between triploid and diploid had not been reported. Most of the earlier study was done to differentiate between fish populations and sex. The study by Bardacki [18] on Nile tilapia, *O. niloticus* using RAPD markers showed that the sex of the fish can be differentiated using genotype profiles produced from PCR amplification of the RAPD markers. In this study, the increment of one chromosome in triploid fish had produced different genotypes, and there was no overlapping of genotype observed. Hence, this may be useful in the identification of the polyploid status of the fishes.

**Figure 1:**
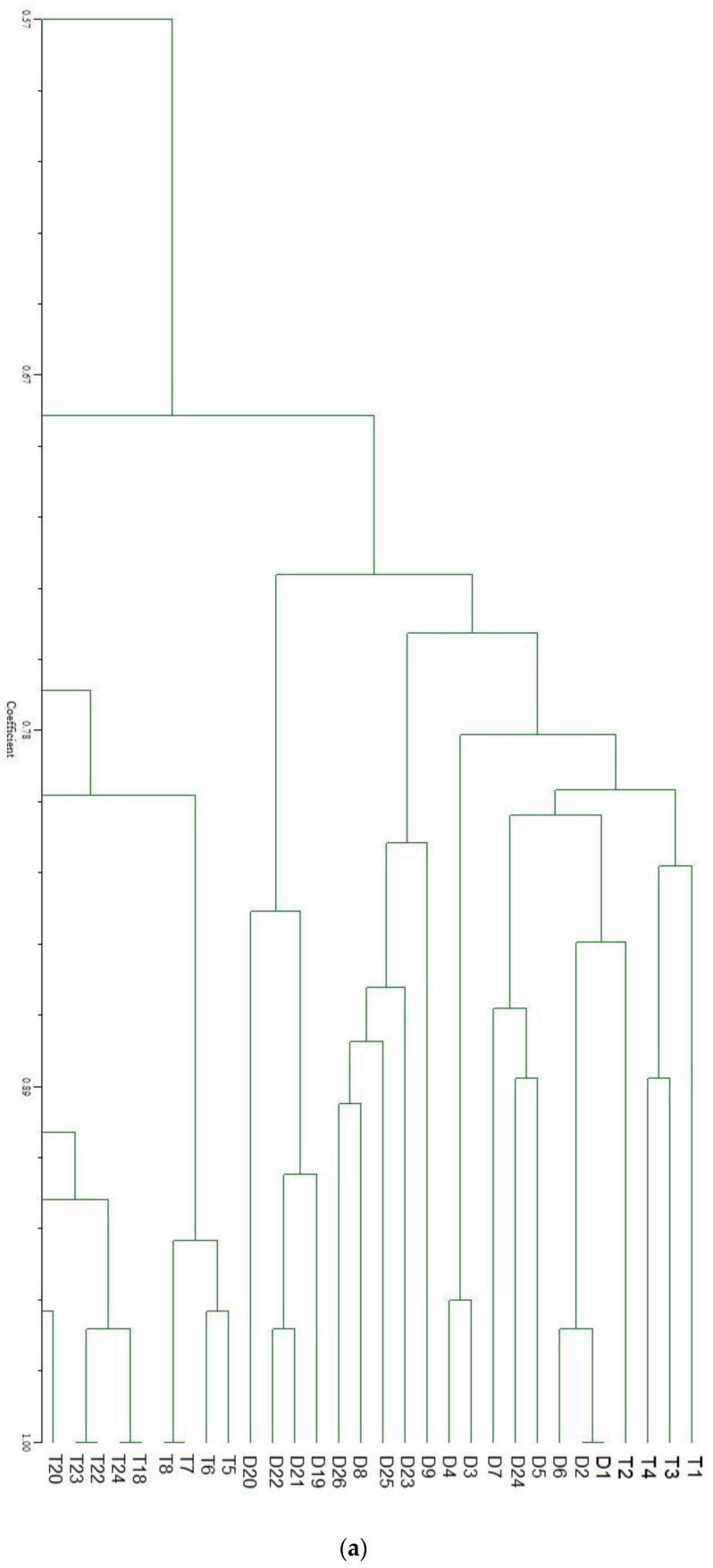

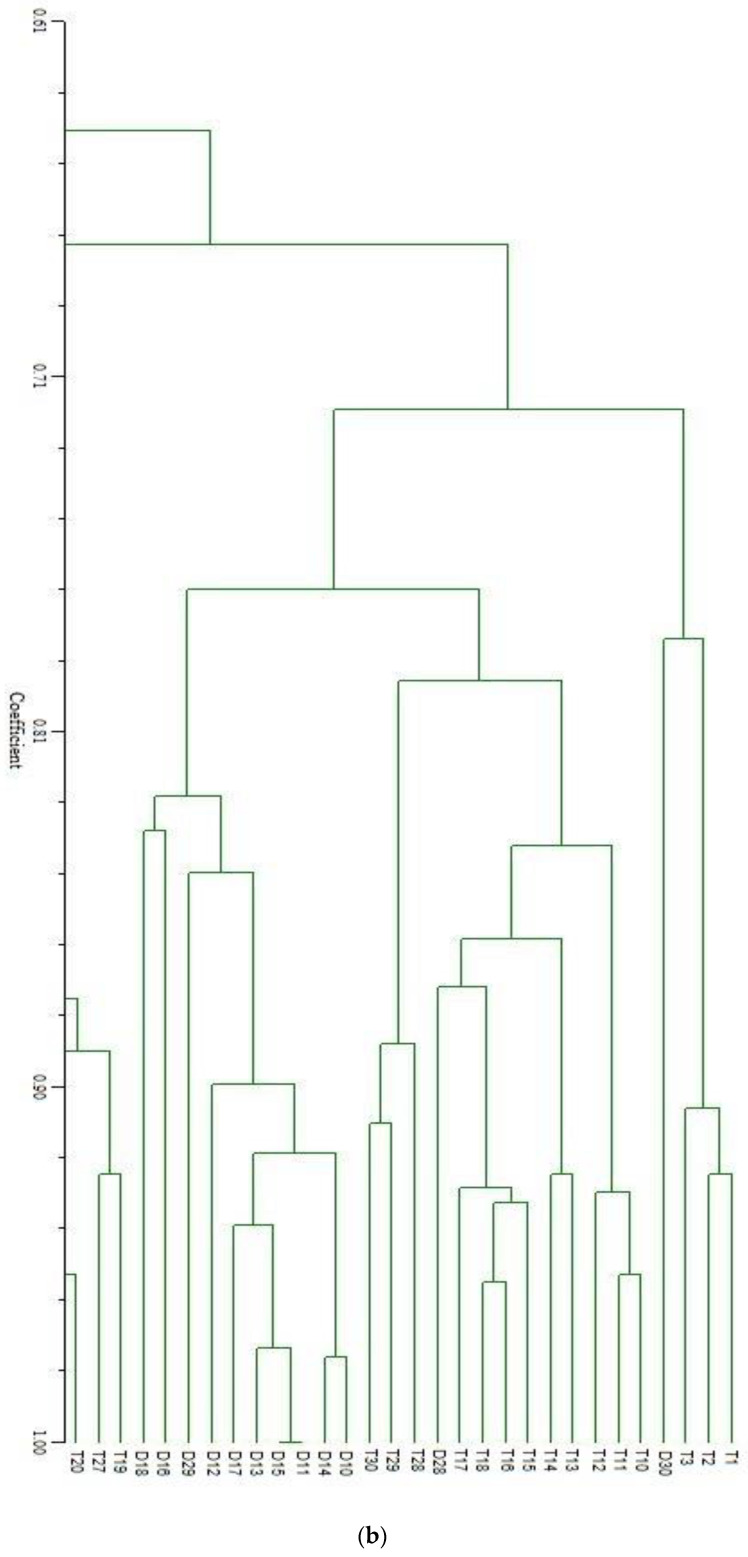

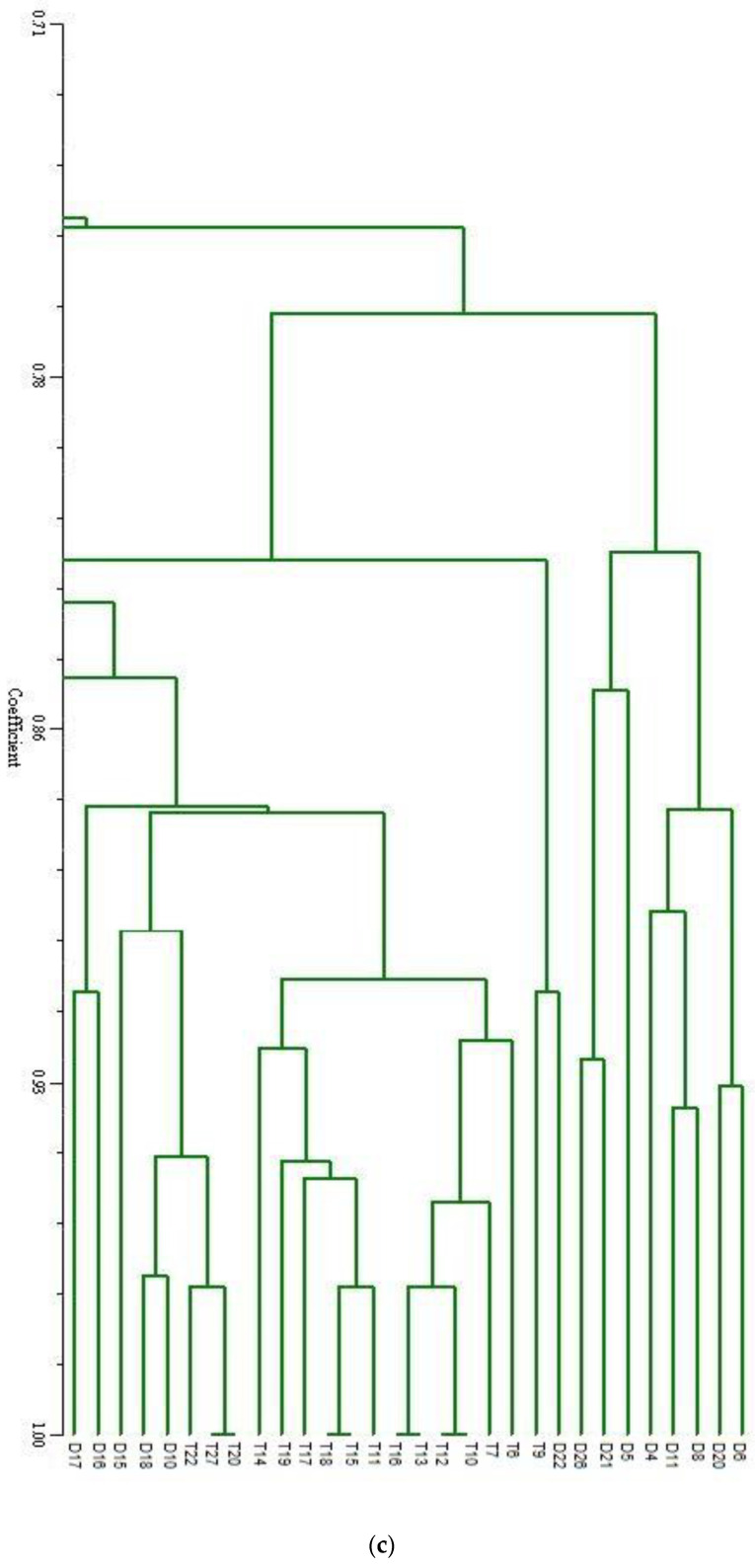
(**a**) The UPGMA dendogram for genetic similarity OPB16. Keys: D = Diploid; T = Triploid. (**b**) The UPGMA dendogram for genetic similarity OPC14. Keys: D = Diploid; T = Triploid. (**c**) The UPGMA dendogram for genetic similarity OPC12. Keys: D = Diploid; T = Triploid.

Generally, a specific genetic marker can be found for certain traits in fish using RAPD markers [17]. Every trait has been hypothesized to have its unique diagnostic marker. However, despite the different genotype observed, no potential diagnostic marker was identified to be usable as a SCAR band from the RAPD analysis in this study. The study by Li et al. [20] found that there were two RAPD-SCAR markers in gift Nile tilapia (*O. niloticus*) which was useful for selection tracking and strain identification. Klinbunga et al. [22] found three RAPD derived SCAR on blue swimming crab *Portunus pelagicus* in Thailand waters. However, in line with the current study, Hatanaka, and Galetti [21] observed no diagnostic bands in *Prochilodus marggravii* from different sampling sites. The lack of a specific fragment for the development of the SCAR marker for triploid and diploid is obviously linked to similar parent stock from which the progenies where gotten and strongly debunk our earlier assumption of a possible genetic change/mutation consequent upon the application of the temperature shock protocol shortly after fertilization. This is justified by the high genetic similarity with wide ranges (0.333–0.976) observed in the study for the three primers used (Table 4). Furthermore, the UPGMA generated dendrogram for triploid and diploid fish was not separated into a distinct cluster, hence displaying an unclear differentiation that grouped the individuals in a fragmented manner (Figure 1). Zhang et al., [29] had earlier opined that progeny from the same broodstock source can contain high genetic similarity. The study by Yue et al. [30] on Asian sea bass (*Lates calcarifer*) showed that the genetic similarity between individuals broodstock ranges between 0 and 0.72 in 170 characterized individuals.

It is clear from this study that the increment in chromosome numbers from the same source of genetic information despite the application of temperature shock did not result in any genetic mutation or differentiation between triploid and diploid individuals. However, this is not the case with a hybrid between two species which showed high genetic differentiation and consequently cluster into unique groups upon an increase or having similar ploidy levels [31]. This is evident in the study by Romana-Eguia et al. [32] who observed that the phylogenetic tree for the Nile tilapia (*O. niloticus*) and Red hybrid tilapia (*O. mossambicus* × *O. niloticus*) clustered into two distinct groups. While triploidy in the current study was caused by an increment of the chromosome from the same species, hybrids are a combination of chromosomes of different species hence the observed differences in genetic diversity reports.

## 4. Conclusions

In conclusion, the 80 RAPD marker used in this study showed no fundamental differences between triploid and diploid African catfish. The choice of RAPD for this study was based on the possibility of identifying a diagnostic marker that can be used as a SCAR marker to detect triploid African catfish. However, future studies can focus on screening other DNA markers for the same purpose. This may include RFLP and AFLP markers which are relatively cheaper DNA markers. While DNA fingerprinting may be a workable/viable alternative to discriminate between the triploid and diploid fish groups because of its robustness, the high-cost implication for this process may discourage its commercial usability as it is not a cheaper alternative. For the time being, erythrocyte characterization using the exclusive triploid range seems to be the easiest, rapid, and cost-effective method of triploid discrimination in fishes.

## Figures and Tables

**Table 1:** Summary of the RAPD profile result of African catfish (*Clarias gariepinus*).

**SN**	**Primers**	**No. of Fragment**	**Fragment Size (bp)**	**Percentage of Polymorphism**	**SN**	**Primers**	**No. of Fragment**	**Fragment Size (bp)**	**Percentage of Polymorphism**
1	OPA-01	14	400–3000	21.43	1	OPB-01	22	300–2200	36.36
2	OPA-02	10	550–2100	60.00	2	OPB-02	20	390–3000	25.00
3	OPA-03	12	450–1500	33.33	3	OPB-03	19	320–2600	52.63
4	OPA-04	6	340–1000	50.00	4	OPB-04	25	200–3000	36.00
5	OPA-05	17	280–2500	41.18	5	OPB-05	20	320–2600	55.00
6	OPA-06	19	250–2700	42.11	6	OPB-06	21	300–2000	38.10
7	OPA-07	20	400–3000	20.00	7	OPB-07	16	180–1400	18.75
8	OPA-08	21	280–2500	42.86	8	OPB-08	20	300–2500	30.00
9	OPA-09	11	250–1700	27.27	9	OPB-09	16	700–2500	43.75
10	OPA-10	15	340–2500	26.67	10	OPB-10	22	350–2300	18.18
11	OPA-11	19	350–3000	36.84	11	OPB-11	23	200–2700	39.13
12	OPA-12	20	250–3000	45.00	12	OPB-12	24	200–2400	25.00
13	OPA-13	15	300–2700	40.00	13	OPB-13	14	370–3000	50.00
14	OPA-14	20	300–1800	30.00	14	OPB-14	14	300–3000	42.86
15	OPA-15	17	350–3000	35.29	15	OPB-15	17	330–1700	23.53
16	OPA-16	21	190–3000	33.33	**16**	**OPB-16**	**13**	**350–2500**	**71.43**
17	OPA-17	21	230–3000	38.10	17	OPB-17	17	300–2000	23.53
18	OPA-18	15	250–2500	46.67	18	OPB-18	17	350–2000	11.76
19	OPA-19	17	200–3000	29.41	19	OPB-19	22	230–2500	50.00
20	OPA-20	17	250–3000	41.18	20	OPB-20	18	300–2700	50.00
**SN**	**Primers**	**No. of Fragment**	**Fragment Size (bp)**	**Percentage of Polymorphism**	**SN**	**Primers**	**No. of Fragment**	**Fragment Size (bp)**	**Percentage of Polymorphism**
1	OPC-01	20	200–2500	35.00	1	OPD-01	20	500–2800	55.00
2	OPC-02	17	300–1700	17.65	2	OPD-02	22	270–2700	45.45
3	OPC-03	17	490–3000	29.41	3	OPD-03	18	350–2300	33.33
4	OPC-04	21	300–2400	23.81	4	OPD-04	16	300–2300	56.25
5	OPC-05	26	200–2500	46.15	5	OPD-05	17	270–2700	58.82
6	OPC-06	19	380–3000	31.58	6	OPD-06	05	400–1100	60.00
7	OPC-07	18	450–2500	27.78	7	OPD-07	15	370–2600	46.67
8	OPC-08	23	220–2500	56.52	8	OPD-08	18	220–1800	27.78
9	OPC-09	17	300–3000	17.65	9	OPD-09	19	350–3000	47.37
10	OPC-10	23	350–2700	21.74	10	OPD-10	22	350–2700	54.55
11	OPC-11	19	320–3000	26.32	11	OPD-11	22	300–3000	45.45
12	OPC-12	19	390–2500	52.63	**12**	**OPD-12**	**20**	**300–3000**	**75.00**
13	OPC-13	20	200–2300	25.00	13	OPD-13	15	470–2200	20.00
**14**	**OPC-14**	**21**	**300–2500**	**61.90**	14	OPD-14	13	300–2500	46.15
15	OPC-15	21	320–2700	38.10	15	OPD-15	19	390–2700	26.32
16	OPC-16	20	210–3000	45.00	16	OPD-16	18	280–2500	33.33
17	OPC-17	18	300–2500	38.89	17	OPD-17	12	410–2300	50.00
18	OPC-18	15	300–1800	20.00	18	OPD-18	18	190–2300	22.22
19	OPC-19	20	300–2000	25.00	19	OPD-19	24	400–2700	41.67
20	OPC-20	20	320–2400	30.00	20	OPD-20	21	250–2500	19.05

Note: Bold and underline primers refer to primers with the highest polymorphic percentage.

**Table 2:** Summary of the RAPD profile results of African catfish (*Clarias gariepinus*) by OPB 16, OPC14, and OPD12.

Primer	Sequence (5′ to 3′)	Number of Fragment	Fragment Size (bp)	Percentage of Polymoprphism (%)	Number of Genotypte
OPB 16	TTTGCCCGGA	20	350–2500	80.00	48
OPC 14	TGCGTGCTTG	35	300–2700	91.40	59
OPD 12	CACCGTATCC	24	350–3000	91.67	52

**Table 3:** (**a**) Genotype number of the triploid and diploid individual as revealed by the primer OPB16. (**b**) Genotype number of the triploid and diploid individual as revealed by the primer OPC14. (**c**) Genotype number of the triploid and diploid individual as revealed by the primer OPC12.

(**a**)																											
Genotype	1	2	3	4	5	6	7	8	9	10	11	12	13	14	15	16	17	18	19	20	21	22	23	24	25		
Triploid	1	1	1	1	1	1									1	1	1	1	1	1	1						
Diploid							1	1	1	1	1	1	1	1								1	1	1	1		
Genotype	26	27	28	29	30	31	32	33	34	35	36	37	38	39	40	41	42	43	44	45	46	47	48				
Triploid			1	1	1	1	1	1	1										1	1							
Diploid	1	1								1	1	1	1	1	1	1	1	1			1	1	1				
(**b**)																											
Genotype	1	2	3	4	5	6	7	8	9	10	11	12	13	14	15	16	17	18	19	20	21	22	23	24	25		
Triploid	1	1	1	1	1	1	1	1	1										1	1	1	1	1	1	1		
Diploid										1	1	1	1	1	1	1	1	1									
Genotype	26	27	28	29	30	31	32	33	34	35	36	36	37	38	39	40	41	42	43	44	45	46	47	48			
Triploid	1										1	1	1	1	1	1	1	1	1	1							
Diploid		1	1	1	1	1	1	1	1	1											1	1	1	1			
Genotype	49	50	51	52	53	54	55	56	57	58	59																
Triploid						1	1	1																			
Diploid	1	1	1	1	1				1	1	1																
(**c**)																											
Genotype	1	2	3	4	5	6	7	8	9	10	11	12	13	14	15	16	17	18	19	20	21	22	23	24	25	26	27
Triploid	1	1	1	1	1	1	1	1	1										1	1	1	1	1				
Diploid										1	1	1	1	1	1	1	1	1						1	1	1	1
Genotype	28	29	30	31	32	33	34	35	36	37	38	39	40	41	42	43	44	45	46	47	48	49	50	51	52		
Triploid						1	1	1	1	1	1										1	1					
Diploid	1	1	1	1	1							1	1	1	1	1	1	1	1	1			1	1	1		

**Table 4:** Genetic similarity of the triploid population, diploid population and between the two populations.

Primer	Triploid Population	Diploid Population	Triploid and Diploid Population
OPB16	0.370–0.969	0.333–0.968	0.348–0.897
OPC14	0.432–0.956	0.457–0.976	0.378–0.913
OPD12	0.667–0.971	0.483–0.968	0.533–0.968

## Data Availability

The data presented in this study are available on request from the corresponding authors. The data are not publicly available due to restrictions from the funding agency.
